# The Principles of Surgery

**Published:** 1852-08

**Authors:** 


*The Principles of Surgery.* By JAMES MILLER, F. R. S. E. F. R. C. S. E., etc., etc., etc. Third American from the second and enlarged Edinburgh Edition. Illustrated by two hundred and forty Engravings on Wood. Revised, with additions, by F. W. SARGENT, M. D., Member of the College of Physicians of Philadelphia, etc., etc. Philadelphia: Blanchard & Lea. 1852. 8vo. pp. 751.

The present edition of this work contains numerous improvements. The subjects have been restudied, and the opinions and doctrines added to, and remodeled wherever recent observations and discoveries have seemed to the author to render expedient; so that "as it now stands," in the language of the American editor, Dr. Sargent, the volume "furnishes the most satisfactory exposition of the modern doctrines on the principles of surgery that is to be found in any single book on the subject in any language." The American editor has contributed, in foot notes, various annotations relating chiefly to inflammation, suppuration, tubercle, cancer, tumours, aneurisms, and anchylosis.

An important improvement upon the previous editions consists in the introduction of a great number of admirable wood engravings, well calculated to explain and illustrate the text.

---

*Obstetrics: the Science and the Art.* By CHARLES D. MEIGS, M. D., Prof. of Midwifery and the Diseases of Women and Children in Jefferson Medical College at Philadelphia, etc., etc., etc. Second edition, revised. With one hundred and thirty-one illustrations. Philadelphia; Blanchard & Lea. 1852. 8vo. pp. 759.

It were insulting to the intelligence of our readers to inform them that, albeit certain extravagances of opinion, among which is to be included a repudiation of chloroform in advance, and eccentricities of diction, the author of this work holds a position as a teacher and practitioner, in which his national brethren have just grounds for pride; and which should command, as it does, for his writings on the science and art of midwifery prompt and extensive circulation.

The first edition of this large work was published three years ago. That edition, although a large one, was exhausted, so that the author was called on to prepare a second which has recently been issued. The latter is considerably augmented, and, as the author states, improved by "recasting some parts, cancelling others, and by an earnest attention to improvements in the literary execution of the whole."

The author apologetically alludes to the fact that his works have been written "amidst the agitations, the distractions and the fatigue of a physician's life." How much is contained in these expressions the physician alone can appreciate.

---

*A Complete Treatise on Midwifery; or the Theory and Practice of Tokology; including the Diseases of Pregnancy, Labor, and the Puerperal State.* By ALF. A. L. M. VELPEAU, M. D. Translated from the French by CHARLES D. MEIGS, M. D., etc., etc., etc. Fourth American, with the additions from the last French edition, by WM. BYRD PAGE, M. D., Lecturer on Obstetrics in the Philadelphia Medical Institute, etc., etc. With numerous illustrations. Philadelphia: Lindsay & Blakiston. 1852. 8vo. pp. 652.

Velpeau's Midwifery translated and edited by Prof. Meigs, has for many years been a standard work in the country. The present edition embraces the latest additions by the author, of course embodying all the modern developments and improvements in the department of medicine of which it treats. It should be added that the task of the revision of the present edition, and comparison with the last French copy, was confided to Dr. Page, with the approbation of the original translator, Prof. Meigs.

The work, in its present form, must prove an acceptable addition to the literature of the subject of which it treats.

---

*The History, Diagnosis, and Treatment of the Fevers of the United States.* By ELISHA BARTLETT, M. D., Professor of Materia Medica and Medical Jurisprudence in the College of Physicians and Surgeons of the University of the State of New York, etc., etc. Third edition, revised. Philadelphia: Blanchard & Lea. 1852. 8vo. pp. 595.

Having noticed at considerable length in this Journal the first and second editions of the above work, we need now but express gratification that so worthy a contribution to the medical literature of this country continues to meet with unabated favor. In justice to the work, and to ourselves, we feel bound to say, what our attentive readers already know, that we have found cause since writing the notices just referred to, for changing our views respecting an important question pertaining to continued fever, viz., the identity or non-identity of typhus and typhoid fever. We were disposed to take issue with Prof. B. on this question when the former editions of this work appeared. As an unforeseen and unexpected result of a careful investigation of the subject by means of the analyses of recorded cases under our own observation, we have been brought to the conclusion, which heretofore we had contended against, viz., that the two varieties of fever just named (typhus and typhoid) are distinct forms of disease. The satisfaction of arriving by logical methods of investigation at the truth, in our estimation far outweighs any gratification of pride in an unchanging consistency of opinion; and it is with real, not affected pleasure, that we declare our conviction of the non-identity of these two forms of fever, a doctrine ably advocated by Prof. B.

---

*Elements of Chemistry; including the Application of the Science in the Arts.* By Thomas Graham, F. R. S., Prof. of Chemistry in University College, London, etc., etc. Second American from an entirely revised and greatly enlarged English Edition. With numerous Wood Engravings. Edited, with notes, by Robert Bridges, Prof. of Chemistry in the Philadelphia College of Pharmacy, etc., etc. Philadelphia: Blanchard & Lea. 1852. 8vo. pp. 331.

The former edition of Graham's Elements of Chemistry has been generally recognized as a valued addition to the text books on this science. In this new edition the size of the work is increased, and the illustrations have been changed in style and nearly doubled in number. The work, in its present form, has been for some time in progress of publication in London in parts, and the entire publication is not yet completed. The American publishers, in consequence of repeated inquiries for the new edition, have issued the first half separately, and the balance may be expected during the present year.

The portion now republished treats of the imponderables, heat, light, etc., and of the elementary substances.

---

*Review of Materia Medica, for the use of Students.* By John B. Biddle, M. D., formerly Prof. of Materia Medica in the Franklin Medical College, of Philadelphia, etc., etc. With illustrations. Philadelphia: Lindsay & Blakiston. 1852. 12 mo. pp. 322.

The author was led to prepare this little volume in consequence of the dearth of elementary works on the Materia Medica proper, text books like the United States Dispensatory, and Preira's Materia Medica, being too voluminous to be conveniently used for that purpose. The chief object of the work is to furnish a "condensed *review* of the elements of materia medica, as a guide to the courses of lectures delivered on this department in the United States." Various illustrations of *indigenous* and *naturalized* plants are introduced.

The work appears to us to be well adapted to fulfill the end for which it was designed.

